# Imaging of proteoglycan and water contents in human articular cartilage with full‐body CT using dual contrast technique

**DOI:** 10.1002/jor.24256

**Published:** 2019-03-28

**Authors:** Miitu K. M. Honkanen, Hanna Matikka, Juuso T. J. Honkanen, Abhisek Bhattarai, Mark W. Grinstaff, Antti Joukainen, Heikki Kröger, Jukka S. Jurvelin, Juha Töyräs

**Affiliations:** ^1^ Department of Applied Physics University of Eastern Finland Kuopio Finland; ^2^ Diagnostic Imaging Center Kuopio University Hospital Kuopio Finland; ^3^ Department of Clinical Radiology Diagnostic Imaging Center Kuopio University Hospital Kuopio Finland; ^4^ Department of Oncology Kuopio University Hospital Kuopio Finland; ^5^ Departments of Biomedical Engineering, Chemistry, and Medicine Boston University Boston Massachusetts; ^6^ Department of Orthopedics, Traumatology and Hand Surgery Kuopio University Hospital Kuopio Finland; ^7^ School of Information Technology and Electrical Engineering The University of Queensland Brisbane Australia

**Keywords:** cartilage, cationic contrast agent, contrast enhanced computed tomography, dual contrast agent, dual energy computed tomography

## Abstract

Assessment of cartilage composition via tomographic imaging is critical after cartilage injury to prevent post‐traumatic osteoarthritis. Diffusion of cationic contrast agents in cartilage is affected by proteoglycan loss and elevated water content. These changes have opposite effects on diffusion and, thereby, reduce the diagnostic accuracy of cationic agents. Here, we apply, for the first time, a clinical full‐body CT for dual contrast imaging of articular cartilage. We hypothesize that full‐body CT can simultaneously determine the diffusion and partitioning of cationic and non‐ionic contrast agents and that normalization of the cationic agent partition with that of the non‐ionic agent minimizes the effect of water content and tissue permeability, especially at early diffusion time points. Cylindrical (*d* = 8 mm) human osteochondral samples (*n* *= *45; four cadavers) of a variable degenerative state were immersed in a mixture of cationic iodinated CA4+ and non‐charged gadoteridol contrast agents and imaged with a full‐body CT scanner at various time points. Determination of contrast agents’ distributions within cartilage was possible at all phases of diffusion. At early time points, gadoteridol, and CA4+ distributed throughout cartilage with lower concentrations in the deep cartilage. At ≥24 h, the gadoteridol concentration remained nearly constant, while the CA4+ concentration increased toward deep cartilage. Normalization of the CA4+ partition with that of gadoteridol significantly (*p *< 0.05) enhanced correlation with proteoglycan content and Mankin score at the early time points. To conclude, the dual contrast technique was found advantageous over single contrast imaging enabling more sensitive diagnosis of cartilage degeneration. © 2019 The Authors. *Journal of Orthopaedic Research* Published by Wiley Periodicals, Inc. J Orthop Res 9999:1–12, 2019.

Osteoarthritis (OA) is the most common joint disease, and causes severe pain and impairment of the joint function. The occurrence of OA is associated with risk factors such as age, genetics, obesity, and/or gender.^1^ OA may also develop after a sudden joint trauma and is then referred to as post‐traumatic osteoarthritis (PTOA).^1^ Although late‐stage OA is irreversible,^2^ development of OA may be prevented or slowed down either surgically or by means of pharmaceutical treatments, if cartilage lesions are detected early enough.^3^, ^4^, ^5^ Unfortunately, accurate and quantitative diagnosis of acute cartilage lesions, bruises, or mechanically compromised tissue is challenging with current standard methods, that is, clinical examination and native X‐ray imaging.^6^, ^7^

The first signs of OA and PTOA‐related changes in cartilage include the loss of proteoglycans (PG), changes in collagen network, and increase in water content.^2^, ^8^, ^9^, ^10^ These changes can be detected using magnetic resonance imaging (MRI).^11^, ^12^, ^13^ MRI has excellent soft tissue contrast, and its clinical use in cartilage imaging has increased. In addition, the improvement in metal artifact reduction techniques has increased the clinical potential of MRI for cartilage imaging in patients with arthroplasty.^14^ However, MRI cannot always be used as a diagnostic tool for OA and PTOA for example due to limited availability, relatively long scan times, and presence of non‐MRI‐compatible foreign bodies.^13^ Hence, it is important to study the diagnostic potential of contrast enhanced computed tomography (CECT) and cone‐beam CT, which are more commonly available and often provide faster scan times and higher spatial resolution (0.1–0.5 mm). Even though CT is based on use of ionizing radiation, new cone‐beam CT scanners provide an image with a low dose.^15^ CECT has been used to evaluate PG content and distribution within cartilage in vitro^16^, ^17^, ^18^ as well as in vivo.^19^, ^20^, ^21^ However, the technique has not yet reached widespread clinical use. Changes in tissue composition and properties influence the diffusion of contrast agents throughout the cartilage. Currently used anionic (negatively charged) contrast agents diffuse into cartilage in inverse proportion to the fixed charge density, that is, concentration of negatively charged PG molecules within cartilage.^22^, ^23^, ^24^

Recently, cationic (positively charged) contrast agents have been introduced for imaging of the articular cartilage.^25^, ^26^ Since the cationic agents are attracted to the negative charge density (PGs) of the tissue, they reach high interstitial concentration at diffusion equilibrium. Thereby, CECT with the cationic contrast agents provides a highly sensitive and direct method to quantify PG distribution and loss.^25^ However, the diffusion of contrast agents is enhanced by increased water and decreased collagen contents as well as disruption of the collagen network organization.^23^, ^27^, ^28^ In early OA, the simultaneous increase in water content and cartilage surface degeneration, and the loss of PGs have opposite effects on the diffusion of cationic contrast agents. This reduces the cationic agent's diagnostic value, especially within a clinically feasible time frame (i.e., ≤1–2 h after contrast agent administration^19^). Furthermore, the increase in water content and cartilage surface degeneration enhance diffusion of all contrast agents, while the PG loss decreases the diffusion of cationic contrast agents.^25^, ^29^, ^30^

As a potential solution to this conundrum, we recently introduced a dual contrast method based on dual energy micro‐CT scanning using a mixture of non‐ionic and cationic contrast agents.^31^ To quantify the concentrations of the two contrast agents in a mixture, measurements are conducted using two different X‐ray energy spectra. This dual energy CT (DECT) scanning method is based on exploiting the element specific *k*‐edges (i.e., photoelectric absorption edges) within the diagnostic energy range.^32^, ^33^ Consequently, the concentrations of an iodinated cationic contrast agent, CA4+, and a non‐ionic gadolinium contrast agent, gadoteridol, in a mixture, can be determined based on the different *k*‐edges (33 and 50 keV for iodine and gadolinium, respectively).

Full‐body DECT is an emerging clinical technique with rapid imaging and advantages in material differentiation as compared to conventional CT systems. DECT scanners are currently used for clinical diagnosis and imaging of gout, heart, urinary tract, and contrast enhanced imaging techniques.^32^, ^34^, ^35^, ^36^, ^37^, ^38^ DECT is performed, for example, using two separate X‐ray tubes and detectors, or one X‐ray tube‐detector pair where the tube voltage is rapidly changed, or one X‐ray tube‐detector pair where the tube voltage is constant and the signal is collected with multilayer detector elements.^32^, ^34^, ^37^, ^38^ The full‐body DECT scanner used in this study (Siemens SOMATOM Definition Flash) is equipped with two X‐ray tubes for dual energy imaging. The present contrast agent mixture is a combination of the cationic iodine‐based contrast agent (CA4+^39^, ^40^), which is highly sensitive to PG distribution, and a clinically used non‐ionic gadolinium‐based contrast agent (gadoteridol, Prohance®), which is sensitive to tissue water content. Since the diffusion of non‐ionic contrast agent into cartilage depends on tissue water content and surface permeability, normalization of the cationic contrast agent partition (i.e., ratio of the agent concentration within cartilage and the immersion bath) with that of non‐ionic agent minimizes the effects of water content and permeability on diffusion of the cationic agent. In principle, this enables determination of the cartilage PG and water contents separately.

The aim of this study is to evaluate the potential of a clinical full‐body DECT scanner for quantitative and spatial assessment of cartilage PG and water contents using the dual contrast technique. Further, for the first time, we determine the diffusion profiles and partitioning of CA4+ and gadoteridol at clinically relevant time points (i.e., ≤2 h) and at diffusion equilibrium (i.e., ≥24 h) using a clinical CT scanner. We hypothesize that simultaneous determination of cationic and non‐ionic contrast agent partitions within cartilage is possible with a full‐body clinical CT scanner. Further, we hypothesize that normalization of the cationic contrast agent partition with that of the non‐ionic agent enhances the cationic agent's ability to detect cartilage PG content, especially at early diffusion time points (1 and 2 h), by reducing the contribution of water content and tissue permeability on diffusion of the cationic agent.

## METHODS

**Figure 1:**
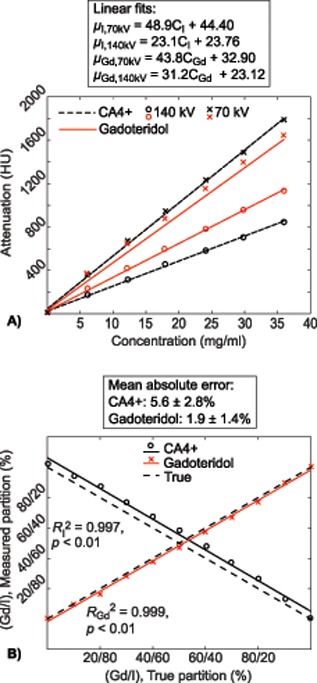
(A) Contrast agent, CA4+ (iodine, I) and gadoteridol (gadolinium, Gd) solutions of different concentrations were imaged separately using 70 and 140 kV tube voltages, to determine mass attenuation coefficients for I (CA4 +) and Gd (gadoteridol). (B) The true and measured contrast agent partitions in contrast agent mixtures determined by using the mass attenuation coefficients derived as the slopes of linear fits in the subfigure A.

CT imaging was conducted using a full‐body dual energy scanner (SOMATOM Definition Flash, Siemens Healthcare, Forcheim, Germany) with 0.098 × 0.098 mm^2^ in‐plane pixel size, 0.5 mm slice thickness, filter type WEDGE_3 (a permanent tube filter equivalent to 6.8 mm of aluminum and a shaped filter (bowtie) equivalent to 0.5 mm of aluminum), and tube voltages of 70 and 140 kV. This energy pair was selected based on the element specific *k*‐edges and was tested with pilot measurements to produce the maximum difference between iodine and gadolinium induced X‐ray attenuation. The dual energy mode could not be used in this study, because the energy pair 70/140 was not available with the present scanner. Thus, the scans were performed sequentially. The total image acquisition time was 1 min (from beginning of the 140 kV scan to end of the 70 kV scan). The mass attenuation coefficients for iodine (*μ*_I,*E*_) and gadolinium (*μ*_Gd,*E*_) were determined by imaging a series of contrast agent solutions with varying concentrations of CA4+ (6.12, 12.24, 18.00, 24.12, 29.88, 36.00 mg I/ml) and gadoteridol (6.12, 12.24, 18.00, 24.12, 29.88, 36.00 mg Gd/ml, Figure 1A). The mass attenuation coefficients were determined from the slopes of the linear fits between the attenuation and concentration values.

The concentrations of the two separate contrast agents in the contrast agent mixture were solved based on Bragg's additive rule for mixtures:

(1) $$\alpha_{E}=C_{I}\cdot \mu_{I,E}+C_{Gd}\cdot \mu_{Gd,E}$$

where *α* is X‐ray attenuation in medium at energy *E* and *C* is the concentration of iodine (I) or gadolinium (Gd) in the mixture. The contrast agent concentrations in the mixture are solved using Equation (1) based on the X‐ray attenuation of two tube voltages (here 70 and 140 kV) as follows:

(2) $$C_{I}=\frac{\alpha_{70kV}\cdot \mu_{Gd,140kV}-\alpha_{140kV}\cdot \mu_{Gd,70kV}}{\mu_{Gd,140kV}\cdot \mu_{I,70kV}-\mu_{Gd,70kV}\cdot \mu_{I,140kV}}$$

(3) $$C_{Gd}=\frac{\alpha_{140kV}\cdot \mu_{I,170kV}-\alpha_{70kV}\cdot \mu_{I,140kV}}{\mu_{Gd,140kV}\cdot \mu_{I,70kV}-\mu_{Gd,70kV}\cdot \mu_{I,140kV}}$$

Prior to the DECT measurements of the human osteochondral samples, the method was evaluated by measuring final contrast agent mixtures with known iodine/gadolinium concentrations of 32.4/3.6, 28.8/7.2, 25.2/10.8, 21.6/14.4, 18.0/18.0, 14.4/21.6, 10.8/25.2, 7.2/28.8 and 3.6/32.4 mg/ml. The concentrations determined using DECT imaging were compared to the known concentrations in the mixtures (Figure 1B).

**Figure 2:**
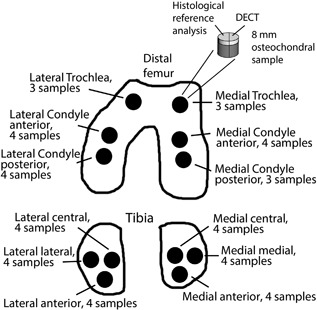
The sample extraction locations and number of samples extracted from each location. The samples (*n* = 45, *d* = 8 mm) were extracted from cadaver (*n* = 4) left and right knee joints. The halves used for histological reference analysis were slightly larger than the halves for the DECT to allow extracting the histological slices from the center of the sample.

A total of 45 osteochondral samples (*d* = 8 mm, Figure 2) were harvested from human cadavers’ [*n* = 4, mean age 71.3 years (ranging from 68 to 79 years)] left and right proximal tibia (*n* = 8) and distal femur (*n* = 8). The Research Committee of the Northern Savo Hospital District, Kuopio University Hospital, Kuopio, Finland had approved the sample collection (decision numbers 58/2013 and 134/2015). The samples were stored frozen (−22°C) and thawed once for another research project (near‐infrared spectroscopy and biomechanical measurements, currently unpublished) before the present experiments. Prior to the DECT scanning, the frozen plugs were halved. One half was prepared for histological reference analysis while the other half was used for the CECT imaging. To ensure that the contrast agent diffusion was only through the articulating surface of the osteochondral sample, the sample sides were carefully coated with a thin layer of cyanoacrylate (Super Glue Precision, Loctite, Düsseldorf, Germany) before immersion in the contrast agent bath.

Cationic contrast agent, CA4+, which is a hydrochloride salt having four positive charges and six iodine atoms (5,5′‐(malonylbis(azanediyl))bis(N^1^,N^3^‐bis(2‐aminoethyl)‐2,4,6‐triiodoisophthalamide, *q* = +4, *M* = 1499.88 g/mol) and non‐ionic contrast agent, gadoteridol (Prohance®, Bracco International B. V., Amsterdam, Netherlands, *q* *= *0, *M* = 558.69 g/mol) were used to prepare the contrast agent mixture. The contrast agents were mixed and diluted with phosphate buffered saline (PBS) including inhibitors of proteolytic enzymes [5 mM ethyleneadiaminetetra‐acetic acid (EDTA; VWR International, France), 5 mM benzamidine hydrochloride hydrate (Sigma–Aldrich Inc., St. Louis, MO)], and penicillin‐streptomycin‐amphotericin B (antibiotic antimycotic solution: 100 U/ml penicillin, 100 μg/ml streptomycin and 0.25 μg/ml amphotericin B, stabilized; Sigma–Aldrich Inc., St. Louis, MO) to prevent tissue degradation. The osmolality of the contrast agent mixture containing 6 mg I/ml (CA4 +) and 18 mg Gd/ml (gadoteridol) was 305 mOsm/kg, as measured using a freeze‐point osmometer (Halbmikro‐osmometer, GWB, Knauer & CO GmbH, Berlin, West‐Germany). The osmolality value was selected to be similar to physiological saline as based on literature this should be more safe in clinical use.^41^

**Figure 3:**
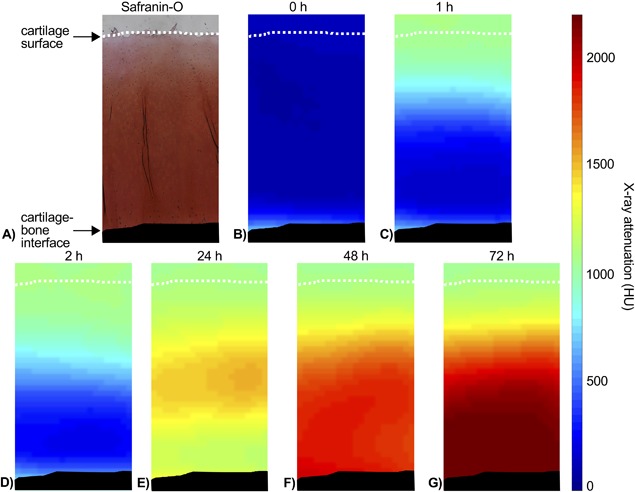
(A) Light microscopic image of Safranin‐O stained cartilage section. (B) CT image of the same cartilage sample immersed in phosphate buffered saline (PBS). (C–G) CT images of the same cartilage sample after immersion in dual contrast agent for 1, 2, 24, 48, and 72 h. Articular surface is marked with a white dashed line and subchondral bone colored with black.

The same imaging protocol was used for the cartilage samples as for the contrast agent solutions and mixtures. The osteochondral samples were first imaged in PBS to acquire non‐contrast images (Figure 3B). Prior to the non‐contrast imaging a piece of play dough was set on the articular surface to aid the segmentation of the cartilage surface from the PBS. Next, the play dough was removed and the osteochondral samples were immersed in a contrast agent mixture (bath volume 100 times cartilage volume). The samples were imaged 1, 2, 24, 48, and 72 h after the immersion in contrast agent (Figure 3C–G). After the second time point (2 h) the contrast agent bath was placed in a refrigerator at 8°C to minimize potential sample degeneration. During the immersion, the bath was gently agitated using a Gyro rocker (STR9 Gyro rocker Platform Rocker, Stuart Scientific, Staffordshire, UK). For imaging the osteochondral samples and the contrast agent phantoms [water, CA4+ (12, 24, 36, 48 mg I/ml) and gadoteridol (6, 12, 18, 24 mg Gd/ml)] were placed onto the treatment table of the CT scanner. For every image acquisition, two different reconstruction kernels (I50s and Q40s) were used. The sharper bone kernel (I50s) was used for segmentation of the articular surface and cartilage‐bone interface as accurately as possible. The neutral and therefore more quantitative kernel (Q40s) was used when analyzing contrast agent diffusion.

Prior the data‐analysis, images acquired at the different time points were co‐registered using 3D Slicer software (version 4.6.2, Kitware, Inc., New York, NY, and Brigham and Women's Hospital, Boston, MA).^42^, ^43^ Subsequently, the articular surface (cartilage‐air interface) and the cartilage‐bone interface were segmented manually from the non‐contrast DECT images using Seg3D software (version 2.2.1, 2015, University of Utah, Salt Lake City, UT). The volume of interest (VOI, 2940 μm × 1960 μm × cartilage thickness) was selected from the center of the cartilage sample by using a custom made MATLAB (R2015b, The MathWorks, Inc., Natick, MA) code.^44^

First, the image stack was interpolated to be isotropic (to straighten the natural curvatures of the articulating surface of cartilage and cartilage‐bone interface). Next, the pixels in VOI were averaged horizontally (approximately 30 × 4 pixels) and the non‐contrast profile was subtracted from the contrast enhanced profiles to determine contrast agent distributions within cartilage. Subsequently, the partition profiles of both contrast agents were calculated (Equations (2) and (3)). Further, the cartilage was divided into zones starting from articular surface to cartilage‐bone interface (0–10, 0–20 … 0–90%). Due to relatively large pixel size (0.098 × 0.098 mm^2^) as compared with cartilage thickness (mean thickness 2.3 mm), the division into smaller zones (10–20%, 20–30%, etc.) was not conducted. Additionally, middle (10–40%) and deep (40–100%) zones of cartilage were analyzed. In analyses, the change in bath concentration induced by contrast agent diffusion into cartilage was taken into account. In addition, CA4+ partition within the cartilage was normalized with that of gadoteridol to determine the CA4+ partition induced by the PG content of cartilage.

The samples sent for histological analysis were decalcified in EDTA. The paraffin embedded samples were then cut into 3 μm thick sections. After the cutting, the paraffin was removed and the sections were stained with Safranin‐O. Safranin‐O is a cationic dye, which is stoichiometrically attracted by negatively charged PGs and therefore reveals the PG distribution in the cartilage.^45^ The optical density (OD, i.e., PG content) was determined with digital densitometry (DD) by analyzing and averaging three sections per sample (Figure 3A). The DD measurements were conducted using a light microscope (Nikon Microphot‐FXA, Nikon CO., Japan) equipped with a monochromatic light source and a 12‐bit CCD camera (ORCA‐ER, Hamamatsu Photonics K.K., Japan). The system was calibrated prior to the actual measurements with neutral density filters (Schott, Germany) with OD range from 0 to 2.3. The articulating surface and cartilage‐bone interface of OD profiles were omitted (10% cut from both ends) to compensate for the partial volume artifact in CT images.

The histological Mankin grading system^46^ was used to evaluate the severity of OA in samples. In the Mankin grading, abnormalities in structure (points from 0 to 6), cellularity (from 0 to 3), Safranin‐O staining (from 0 to 4), and tidemark integrity (from 0 to 1) were evaluated. Three Safranin‐O stained sections per sample were scored. All sections of samples were blind coded and scored in random order. The final score for each section was calculated as the average of four independent assessors (M. Honkanen, N. Hänninen, M. Prakash and R. Shaikh). The score for each sample was calculated as an average of the three sections.

The relationship between the measured and true CA4+ and gadoteridol concentrations in the contrast agent mixture phantoms was evaluated using Pearson correlation analysis. The non‐normality of the measured osteochondral sample data were confirmed with the Shapiro‐Wilk test. Therefore, the statistical significance of dependence between the contrast agent partitions, OD, and the Mankin score were determined using Spearman's correlation. In addition, the statistical significance of difference between these correlation coefficients was tested with bootstrapping (using 10,000 simulations). All of the statistical analyses were conducted by M. Honkanen using SPSS (v. 21.0 SPSS Inc. and v. 25.0 SPSS Inc., IBM Company, Armonk, NY).

## RESULTS

The contrast agent mixture compositions, determined with DECT, correlated linearly with the true mixture compositions (for iodine *R* ^2^ = 0.997, *p *< 0.01 and for gadolinium *R* ^2^ = 0.999, *p *< 0.01), with mean absolute errors of 5.6 ± 2.8% and 1.9 ± 1.4% for iodine and gadolinium concentrations, respectively (Figure 1B).

**Figure 4:**
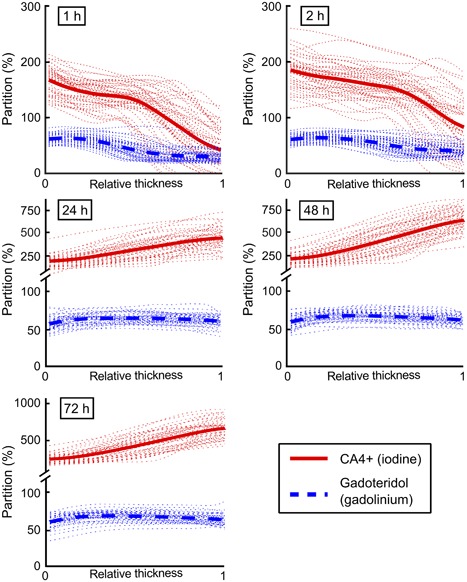
Sample specific contrast agent partition profiles (*n* = 45, dotted lines) and mean partition profiles for CA4+ (iodine, solid lines) and gadoteridol (gadolinium, dashed lines) at 1, 2, 24, 48, and 72 h after immersion in the dual contrast agent mixture. In horizontal axis, 0 denotes the articular surface and 1 the cartilage‐bone interface. Please, note axis break and different scaling on vertical axis.

The mean bulk contrast agent partitions in cartilage increased from 112% (at 1 h) to 442% (at 72 h) for CA4+, and from 44% (at 1 h) to 65% (at 72 h) for gadoteridol (Table 1) as a function of immersion time. At the early time points (1 and 2 h), the contrast agent partitions decreased (Figure 4) along the cartilage depth, and, at later time points (24, 48, and 72 h) increasing and decreasing trends for CA4+ and gadoteridol, respectively, were revealed.

**Table 1:** The Bulk (Average Along the Whole Cartilage Thickness) Partitions of CA4+ (Iodine) and Gadoteridol (Gadolinium) in Articular Cartilage at 1, 2, 24, 48, and 72 h After the Contrast Agent Immersion, Optical Densities (i.e., Proteoglycan Content), and Mankin Scores for Cartilage Samples from Different Anatomical Sites

	Contrast agent partition (%) (normalized partition for CA4 +)											
	1 h		2 h		24 h		48 h		72 h			
Location	CA4+	Gadoteridol	CA4+	Gadoteridol	CA4+	Gadoteridol	CA4+	Gadoteridol	CA4+	Gadoteridol	Optical density	Mankin score
FLC	110 ± 20	44 ± 8	142 ± 15	51 ± 6	324 ± 61	64 ± 3	386 ± 76	66 ± 7	422 ± 84	67 ± 5	0.85 ± 0.19	4.86 ± 2.14
(*n* = 8)	(261 ± 74)		(289 ± 51)		(511 ± 112)		(602 ± 162)		(649 ± 172)			
FMC	120 ± 16	46 ± 10	152 ± 23	53 ± 9	376 ± 49	60 ± 6	428 ± 63	65 ± 4	439 ± 77	69 ± 8	0.87 ± 0.07	6.45 ± 1.99
(*n* = 7)	(292 ± 81)		(302 ± 79)		(642 ± 98)		(671 ± 109)		(652 ± 176)			
FT	122 ± 18	42 ± 8	150 ± 12	53 ± 7	368 ± 62	61 ± 4	426 ± 108	62 ± 4	452 ± 113	63 ± 4	0.89 ± 0.20	4.96 ± 1.08
(*n* = 6)	(313 ± 90)		(289 ± 50)		(615 ± 136)		(709 ± 225)		(729 ± 189)			
TLC	105 ± 29	43 ± 13	141 ± 33	50 ± 9	320 ± 60	65 ± 6	398 ± 88	65 ± 5	429 ± 91	65 ± 4	0.81 ± 0.21	5.77 ± 1.78
(*n* = 12)	(247 ± 79)		(290 ± 80)		(506 ± 120)		(639 ± 188)		(672 ± 155)			
TMC	112 ± 37	44 ± 4	142 ± 39	53 ± 8	353 ± 85	62 ± 7	426 ± 87	63 ± 7	464 ± 91	62 ± 6	0.87 ± 0.15	6.62 ± 1.14
(*n* = 12)	(245 ± 93)		(273 ± 83)		(599 ± 222)		(700 ± 201)		(764 ± 176)			
Total	112 ± 28	44 ± 9	144 ± 29	52 ± 8	345 ± 70	63 ± 6	412 ± 87	64 ± 6	442 ± 93	65 ± 6	0.85 ± 0.18	5.83 ± 1.81
(*n* = 45)	(265 ± 88)		(287 ± 73)		(567 ± 162)		(663 ± 187)		(697 ± 178)			

FLC, femoral lateral condyle; FMC, femoral medial condyle; FT, femoral trochlea; TLC, tibial lateral condyle; TMC, tibial medial condyle.

The CA4+ and gadoteridol partitions within cartilage could be simultaneously determined with a clinical full‐body CT scanner at the early phase of diffusion as well as at later diffusion time points. The normalized CA4+ partition correlated significantly (*p *< 0.05) with OD (i.e., PG content) in all cartilage zones from 0 to 90% at all diffusion time points (Table 2). Moreover, the normalized CA4+ partition in all cartilage zones from 0 to 90% and Mankin score were significantly related (*p *< 0.05) at 1, 2, 48, and 72 h time points (Table 3). The normalized bulk CA4+ partition correlated significantly (*p *< 0.05) with OD at near diffusion equilibrium (24, 48, and 72 h) and with the Mankin score at 1, 2, and 48 h time points. Although non‐normalized CA4+ partition correlated significantly (*p *< 0.05) with OD at the 1 h (zones 0 to 50%) and 2 h (0 to 70%) time points, the correlation coefficients (*ρ*) were lower than those for normalized CA4+ partition (Table 2). Importantly, the normalization improved significantly (*p *< 0.05) the correlation between CA4+ partition in all cartilage zones (0–10 … 0–100, 10–40, and 40–100%) and Mankin score at the 1 and 2 h time points [except in the deep layer (40–100%) for 1 h and in the superficial layers (0–10 and 0–30%) for 2 h]. Moreover, the normalization enhanced the correlation between CA4+ partition and OD significantly (*p *< 0.05) at the 1 h time point in the middle and deep zones (0–20 … 0–90% and 10–40%). In addition, the non‐normalized CA4+ partition did not correlate with the Mankin score at the early time points (Table 3). The bulk CA4+ partition correlated significantly (*p *< 0.05) with OD and Mankin score at the later time points (24, 48, and 72 h, and 48 and 72 h, respectively; Tables 2 and 3). Gadoteridol partition in all cartilage zones (except the deep zone, 40–100%, with OD) correlated significantly (*p *< 0.05) with OD and Mankin score at the early time points (1 and 2 h, Tables 2 and 3). Near the diffusion equilibrium (at 48 h) the gadoteridol partition correlated significantly (*p *< 0.05) in the middle and deep zones with OD and Mankin score.

**Table 2:** Spearman's Correlation Coefficients (*ρ*) Between Optical Density (OD) and CA4+, Normalized CA4+, and Gadoteridol Partitions Within Different Zones from Articular Surface (0%) to Whole Cartilage Thickness (100%, Bulk) at All Diffusion Time Points

	Superficial				Middle						Deep	Bulk
OD	0–10%	0–20%	0–30%	0–40%	10–40%	0–50%	0–60%	0–70%	0–80%	0–90%	40–100%	0–100%
**1 h**												
CA4+	0.445**	0.406**	0.376*	0.352*	0.230	0.321*	0.275	0.222	0.144	0.112	0.058	‐0.009
CA4+ normalized	0.573**	0.562**	0.595**	0.597**	0.505**	0.586**	0.576**	0.519**	0.452**	0.399**	0.240	0.284
Gadoteridol	−0.581**	−0.566**	−0.564**	−0.545**	−0.527**	−0.526**	−0.502**	−0.457**	−0.432**	−0.409**	−0.211	−0.422**
**2 h**												
CA4+	0.347*	0.356*	0.364*	0.392**	0.345*	0.395**	0.364*	0.329*	0.247	0.207	0.052	0.055
CA4+ normalized	0.457**	0.444**	0.450**	0.469**	0.442**	0.498**	0.481**	0.461**	0.396**	0.331*	0.006	0.232
Gadoteridol	−0.469**	−0.458**	−0.469**	−0.488**	−0.456**	−0.492**	−0.425**	−0.411**	−0.352*	−0.309*	−0.060	−0.336*
**24 h**												
CA4+	0.520**	0.518**	0.562**	0.577**	0.594**	0.594**	0.584**	0.575**	0.562**	0.564**	0.526**	0.608**
CA4+ normalized	0.432**	0.454**	0.479**	0.530**	0.510**	0.558**	0.547**	0.523**	0.515**	0.531**	0.499**	0.544**
Gadoteridol	−0.268	−0.251	−0.265	−0.267	−0.203	−0.266	−0.276	−0.263	−0.256	−0.264	−0.208	−0.272
**48 h**												
CA4+	0.590**	0.569**	0.589**	0.608**	0.648**	0.616**	0.598**	0.588**	0.579**	0.558**	0.546**	0.621**
CA4+ normalized	0.578**	0.562**	0.584**	0.607**	0.621**	0.614**	0.601**	0.563**	0.543**	0.497**	0.473**	0.525**
Gadoteridol	−0.380*	−0.367*	−0.414**	−0.444**	−0.381**	−0.459**	−0.479**	−0.440**	−0.390**	−0.332*	−0.181	−0.274
**72 h**												
CA4+	0.472**	0.491**	0.535**	0.543**	0.638**	0.593**	0.583**	0.587**	0.579**	0.562**	0.582**	0.646**
CA4+ normalized	0.458**	0.434**	0.492**	0.512**	0.575**	0.552**	0.542**	0.530**	0.518**	0.473**	0.480**	0.533**
Gadoteridol	−0.242	−0.205	−0.232	−0.291	−0.237	−0.303*	−0.269	−0.212	−0.201	−0.116	−0.013	−0.091

**p < 0.01 and *p < 0.05.

**Table 3:** Spearman's Correlation Coefficients (*ρ*) Between Mankin Score and CA4+, Normalized CA4+, and Gadoteridol Partitions Within Different Zones from Articular Surface (0%) to Whole Cartilage Thickness (100%, Bulk) at All Diffusion Time Points

	Superficial				Middle						Deep	Bulk
Mankin score	0–10%	0–20%	0–30%	0–40%	10–40%	0–50%	0–60%	0–70%	0–80%	0–90%	40–100%	0–100%
1 h												
CA4+	−0.251	−0.247	−0.237	−0.219	−0.184	−0.195	−0.165	−0.094	0.019	0.088	0.282	0.169
CA4+ normalized	−0.435**	−0.439**	−0.474**	−0.529**	−0.516**	−0.546**	−0.580**	−0.557**	−0.492**	−0.423**	−0.269	−0.363*
Gadoteridol	0.556**	0.585**	0.598**	0.609**	0.606**	0.628**	0.666**	0.687**	0.693**	0.698**	0.614**	0.687**
2 h												
CA4+	−0.185	−0.219	−0.216	−0.212	−0.212	−0.215	−0.225	−0.203	−0.136	−0.067	0.139	0.007
CA4+ normalized	−0.325*	−0.343*	−0.342*	−0.372*	−0.360*	−0.399**	−0.436**	−0.482**	−0.454**	−0.420**	−0.351*	−0.363*
Gadoteridol	0.436**	0.452**	0.462**	0.496**	0.484**	0.525**	0.550**	0.601**	0.589**	0.593**	0.556**	0.575**
24 h												
CA4+	−0.356*	−0.342*	−0.374*	−0.379*	−0.409**	−0.403**	−0.396**	−0.378*	−0.340*	−0.292	−0.162	−0.232
CA4+ normalized	−0.232	−0.214	−0.234	−0.268	−0.283	−0.284	−0.288	−0.276	−0.256	−0.224	−0.152	−0.179
Gadoteridol	0.089	0.036	0.040	0.033	0.014	0.047	0.064	0.086	0.087	0.086	0.084	0.062
48 h												
CA4+	−0.509**	−0.507**	−0.508**	−0.506**	−0.495**	−0.491**	−0.481**	−0.472**	−0.454**	−0.413**	−0.275	−0.349*
CA4+ normalized	−0.464**	−0.464**	−0.478**	−0.484**	−0.466**	−0.485**	−0.473**	−0.463**	−0.437**	−0.416**	−0.321*	−0.386**
Gadoteridol	0.288	0.272	0.304*	0.298*	0.276	0.318*	0.343*	0.342*	0.345*	0.342*	0.360*	0.347*
72 h												
CA4+	−0.406**	−0.445**	−0.469**	−0.481**	−0.483**	−0.470**	−0.465**	−0.457**	−0.449**	−0.423**	−0.282	−0.367*
CA4+ normalized	−0.377*	−0.349*	−0.374*	−0.380**	−0.391**	−0.394**	−0.398**	−0.399**	−0.377*	−0.341*	−0.220	−0.257
Gadoteridol	0.151	0.146	0.124	0.140	0.087	0.160	0.171	0.158	0.144	0.099	0.016	0.061

**p < 0.01 and *p < 0.05.

## DISCUSSION

For the first time, we use a clinical full‐body DECT scanner for dual contrast imaging of articular cartilage. Based on the present results, DECT enables simultaneous determination of the contrast agents (CA4+ and gadoteridol) distributions within cartilage at diffusion equilibrium but also at the early clinically relevant diffusion time points. Furthermore, the normalization of the CA4+ partition with that of gadoteridol significantly (*p *< 0.05) improves the ability of the cationic agent to detect cartilage PG content (i.e., OD) at early diffusion time points (1 and 2 h).

Currently, contrast enhanced CT imaging is most often conducted using anionic iodinated agents (most commonly ioxaglate). However, in theory, cationic contrast agents are diagnostically more sensitive.^26^ In contrast to anionic contrast agents, cationic agents are attracted by negatively charged PGs, and hence their partitioning is directly proportional to the PG distribution within cartilage. Thus, the use of cationic agents affords improved contrast and yields greater capabilities to detect PG content and its variation within articular cartilage.^25^, ^26^ As the diffusion of CA4+ is controlled by the PG and water contents within cartilage, especially at early stage of diffusion, the separation between healthy (high PG content) and degenerated (low PG content and elevated water content) cartilage can be challenging due to similar CA4+ uptake and hence, could lead to misinterpretation of the PG distribution. On the other hand, diffusion of gadoteridol is controlled by the water content and permeability (surface degeneration) of cartilage. Hence, normalization of the CA4+ partition in cartilage with that of gadoteridol may increase the accuracy for detecting PG loss. In this study, the components of the dual contrast agent were CA4+ and gadoteridol. However, the dual contrast technique is applicable with combination of other contrast agents as long as separation between the *k*‐edge energies of the agent components is large enough.

Both contrast agent partition profiles decrease as a function of cartilage depth at the early time points. In contrast, at and after 24 h of diffusion the gadoteridol partition remains relatively constant with a small decrease in the mean profile from the highest value at the superficial layer of 66 ± 2% to cartilage‐bone interface 61 ± 1%. The average CA4+ partition increases from 112% (at 1 h) up to 442% (at 72 h). This increase in the average partition and the increasing CA4+ partition throughout the cartilage thickness are in line with earlier studies.^26^, ^31^, ^47^, ^48^ The increasing partition of CA4+ in deep cartilage is due to the higher PG content.^49^, ^50^ The gadoteridol partition decreases towards the deep cartilage at all diffusion time points, as expected. This result is due to the decreasing cartilage water content along the cartilage depth.^23^, ^27^, ^51^, ^52^

A statistically significant relation (*p *< 0.05) is found between the CA4+ partition and proteoglycan content (i.e., OD) at all diffusion time points (Table 2), except for the early time points (1 and 2 h) in the deep cartilage. During the first two hours, the diffusion of the contrast agents has not yet reached the full depth of cartilage, which explains this lack of correlation. Along our hypothesis, the normalization of CA4+ partition with that of gadoteridol provides statistically significantly (*p *< 0.05) higher correlation coefficients when compared to non‐normalized CA4+ at the early time points (especially at 1 h). However, the normalization of CA4+ did not improve the correlation at time points close to the diffusion equilibrium (24, 48, and 72 h), and no correlation was found between the gadoteridol partition and proteoglycan content at the 24 and 72 h time points. This result may be due to the high CA4+ concentration in the deep cartilage near the diffusion equilibrium, which causes X‐ray beam hardening and overestimation of gadoteridol concentration. Alternatively, the variation in gadoteridol concentration in late diffusion time points and in deep cartilage is rather small when compared to that in CA4+ concentration. This could explain why the normalization did not improve the correlations near the diffusion equilibrium. In contrast, at the beginning of the diffusion (1 and 2 h after immersion) the contrast agent flux into cartilage is high and affected by the tissue water content and surface permeability.

At the early diffusion time points Spearman's correlation coefficients (*ρ*) between normalized CA4+ partition, and OD (PG content) and Mankin score are higher than those between non‐normalized CA4+ partition, and OD and Mankin score (Tables 2 and 3). As our clinical interest lie with diagnosis of early PTOA, contrast agent diffusion in the cartilage superficial and middle zones at the early time points are especially relevant. The correlations between normalized CA4+ partition and cartilage PG content [0.23 < *ρ *< 0.54 for full thickness and 0.33 < *ρ < *0.61 in different zones (0–10, 0–20 … 0–90%) at all diffusion time points] are lower than those reported between normalized CA4+ partition and biomechanical properties [*ρ* ≈ 0.75 for cartilage surface (top 500 μm) at equilibrium] in our earlier micro‐CT study.^31^ This is likely mainly due to a lower signal‐to‐noise ratio and the spatial resolution of the clinical CT scanner compared to a micro‐CT scanner. However, with continued development of clinical dual energy CT systems, these limitations may be minimized in the future.

Although MRI is a routine method for joint imaging, the present results indicate that the dual contrast CT imaging might provide a feasible alternative. However, it must be noted that MRI is a non‐invasive imaging method for diagnosing cartilage degeneration while CECT requires contrast agent injection and use of ionizing radiation. Then again, CT possesses higher spatial resolution, shorter imaging times, lower costs, and better availability than MRI. Furthermore, with CECT, early changes in cartilage condition are detected quantitatively after acute injury as well as changes in the subchondral bone.^21^, ^53^

As a first study describing dual contrast measurements of human articular cartilage during dynamic diffusion, this study also has several limitations. First, when the partitions are calculated using two image acquisitions with different energies, the assumption is that both acquisitions are conducted simultaneously. This was nearly possible with the applied clinical full‐body DECT scanner, since the total image acquisition time was only ≈1 min. Therefore, the error caused by the diffusion is assumed to be minimal. However, this is not the case when this technique is applied using high resolution micro‐CT devices with long acquisition times. It should be noted that at diffusion equilibrium, this error induced by diffusion is almost non‐existent. Second, the relatively large voxel size of full‐body DECT images combined with partial volume artifact, related to pixel loss at the cartilage surface, might have caused minor but systematic errors in detection of cartilage interfaces (Figure 3). However, based on the present study the resolution of the full‐body DECT is sufficient for determination of contrast agent partitions in human cartilage. Third, and the main limitation of this study is the relatively low number (*n* = 4; eight knees) of cadavers. Hence, several samples were extracted from each knee joint causing dependency between the samples. In addition, samples were from different locations (from tibia and femur as well as from medial and lateral side) causing variation in structural and compositional properties of samples. Furthermore, the samples experienced two freeze‐thaw cycles before experiment, which might have caused minor degeneration but should have a negligible effect on the present results.^54^, ^55^, ^56^

Based on these encouraging results, further in vitro, pre‐clinical, and clinical studies with larger pools of samples/animals/patients are needed to reveal the diagnostic potential of DECT. In conclusion, the introduced dual contrast method combined with clinical full‐body DECT enables simultaneous estimation of the cartilage water (gadoteridol) and PG (CA4+) contents, and, therefore, is of potential clinical diagnostic use.

## AUTHOR CONTRIBUTION

MKM Honkanen, H Matikka, JTJ Honkanen, A Bhattarai, JS Jurvelin and J Töyräs planned the study protocol and conception. A Joukainen and H Kröger applied for ethical approval, participated in the sample extraction and gadoteridol purchase. CA4+ contrast agent was prepared in the laboratory of MW Grinstaff. DECT data acquisition was done by MKM Honkanen and H Matikka. MKM Honkanen conducted the digital densitometry measurements and the data‐analysis. Data interpretation was done by MKM Honkanen, JTJ Honkanen, A Bhattarai and J Töyräs. MKM Honkanen, JTJ Honkanen, MW Grinstaff, JS Jurvelin and J Töyräs drafted the manuscript. All the authors have read and approved the final submitted manuscript.
